# How inclusive were strategies to prevent the spread of COVID-19 for people with disabilities? Evidence from qualitative research in eight low- and middle-income countries

**DOI:** 10.1186/s12939-025-02482-7

**Published:** 2025-05-05

**Authors:** Xanthe Hunt, Sarah Marks, Shaffa Hameed, Donruedee Srisuppaphon, Francisco Diez-Canseco, Wachara Riewpaiboon, Shaheda Viriyathorn, Viroj Tangcharoensathien, Divya Goyal, Tracey Smythe, Rifat Shahpar Khan, Luong Anh Ngoc, John Ganle, Shailaja Tetali, Lopita Huq, Tom Shakespeare, Zeynep Ilkkursun, Ceren Acarturk, Vu Quynh Mai, Lena Morgon Banks

**Affiliations:** 1 Africa Health Research Institute (AHRI), Nelson R. Mandela School of Medicine 3rd Floor, K-RITH Tower Building, 719 Umbilo Rd, Durban, Umbilo 4001 South Africa; 2 Mental Health, Alcohol, Substance Use, and Tobacco Research Unit (MAST-RU), South African Medical Research Council (SAMRC), Cape Town, South Africa; 3 International Centre for Evidence in Disability (ICED), Department of Population Health, London School of Hygiene and Tropical Medicine, London, UK; 4 Ministry of Public Health, Sirindhorn National Medical Rehabilitation Institute, Nonthaburi, Thailand; 5 CRONICAS Center of Excellence in Chronic Diseases, Universidad Peruana Cayetano Heredia, Lima, Peru; 6 Faculty of Medicine, Ramathibodi Hospital, Mahidol University, Bangkok, Thailand; 7 International Health Policy Program, Ministry of Public Health, Nonthaburi, Thailand; 8 Division of Physiotherapy, Department of Health and Rehabilitation Sciences, Stellenbosch University, Cape Town, South Africa; 9 BRAC Institute of Governance & Development, Dhaka, Bangladesh; 10 Center for Training and Research On Substance Abuse - HIV, Hanoi Medical University, Hanoi, Viet Nam; 11 School of Public Health, University of Ghana, Legon, Accra, Ghana; 12 Indian Institute of Public Health, Hyderabad, India; 13 Department of Psychology, Koc University, Istanbul, Türkiye; 14 Center for Population Health Science, Hanoi University of Public Health, Hanoi, Viet Nam

**Keywords:** COVID- 19, Prevention, Pandemic, Disability, Emergency response

## Abstract

**Background:**

From the outset of the pandemic there were calls to ensure people with disabilities were included in prevention and response measures, given their increased risk of health consequences from COVID-19 infection. This study sought to explore people with disabilities’ experiences of inclusion in the response to the COVID-19 pandemic, to understand how such responses can be more inclusive in the future.

**Methods:**

Qualitative interviews were conducted with 372 people with disabilities and their caregivers in Bangladesh, Ghana, India, Peru, Thailand, Türkiye (with Syrian refugees), Viet Nam, and Zimbabwe between 1 December 2020 and 28 February 2023, and analysed using thematic analysis.

**Results:**

The study found that people with disabilities demonstrated high levels of knowledge about COVID-19 and were willing to adhere to prevention measures. However, participants noted that countries’ COVID-19 responses were largely not inclusive of people with disabilities; that pandemic information was seldom available in accessible formats; and that adhering to social distancing and other mandates was challenging and incurred personal and economic costs.

**Conclusions:**

Consequently, the pandemic compounded existing barriers and inaccessibility experienced by people with disabilities and contributed to inequality.

## Background

The Coronavirus Disease- 2019 (COVID- 19) pandemic was an unprecedented global health crisis, with over 624 million cases worldwide, and over 7 million deaths as of November 2024 (Center for Systems Science and Engineering (CSSE) at Johns Hopkins University, 2022). Complications such as post-COVID- 19 conditions (‘long COVID’) –has a global prevalence of 0.43 (95% CI: 0.39,0.46) – and are worsening population health and health systems’ strain [8]. In the acute phase of the pandemic, between 2020–2021, certain groups of people experienced a heightened risk of COVID- 19, both in terms of risk of infection and of severe health outcomes if infected [16, 58]. This included people with disabilities, who were at disproportionate risk of serious consequences from COVID- 19 infection [26, 32, 34, 52]. Given the additional challenges and barriers to health and social care experienced by people with disabilities in low- and middle-income countries (LMICs), it is likely that they experienced additional vulnerabilities.

Studies in the United States found that the odds of COVID- 19 diagnosis increased by 20 percent for people with disabilities [15], and incidence and hospitalization rates were higher for this population [59]. Furthermore, research shows that people with certain disabilities, namely autism spectrum conditions, intellectual disabilities, chronic respiratory conditions, and mental health conditions, experienced higher risk of COVID- 19 infection and severe symptoms [10, 14], Gleason et al. [34, 39, 47]. A study using the international TriNetX COVID- 19 Research Network platform showed that younger people with intellectual disabilities experienced higher fatality rates [52]. People in the United Kingdom whose disabilities affected their daily functioning were three times more likely to have died from COVID- 19 than individuals without limited functioning [6]. This elevated risk of infection and mortality among people with disabilities results from a range of factors including pre-existing medical conditions, age and living in residential facilities [25, 30, 33, 41, 47, 57]. Additionally, inequalities in socioeconomic circumstances that pre-dated the COVID- 19 pandemic heightened the risk of infection and more severe outcomes from COVID- 19, including barriers to accessing healthcare and information, heightened risk of poverty, inadequate housing and restricted autonomy, compounded [54].

Despite evidence of heightened risks and vulnerabilities, strategies to prevent the spread of COVID- 19 and improve access to care lacked inclusivity for people with disabilities [27, 49]. For instance, West African policy responses to COVID- 19 failed to take into account the lived experience and priorities of people with disabilities [1]. Similarly, analysis of policies in four South American countries found only one country (Peru) voted for specific legislation to protect the rights of people with disabilities while others only produced recommendations [45]. Many of these recommendations put the onus on people with disabilities to protect themselves during the pandemic (e.g., through shielding), without providing them with the resources and guidance to make it feasible. A scoping review of articles on the prevention of COVID- 19 among people with intellectual and developmental disabilities highlighted that they faced various barriers to adhering to prevention measures, including, high costs for obtaining personal protective equipment (PPE) [50].

Emergence from the most acute phase of the pandemic, there is increasing recognition that pandemics and other shocks related to climate change, conflict, and natural disasters, are to become a familiar feature of the global landscape [19]. While continued COVID- 19 cases necessitate reflection on strengths and areas for improvement prevention, future pandemic and crisis preparedness can be informed by analysis of the COVID- 19 pandemic occurrences. Without an accurate understanding of the COVID- 19 prevention measures experienced by people with disabilities, they face the risk of repeated future exclusion. This study sought to explore people with disabilities’ experiences of inclusion in the response to the COVID- 19 pandemic, with a view to understanding how such responses can be made more inclusive in the future.

## Methods

In-depth interviews were conducted with people with disabilities and/or their caregivers in Bangladesh, Ghana, India, Thailand, Türkiye (with Syrian refugees), Viet Nam, and Zimbabwe. These interviews were typically one-to-one, unless a respondent required additional support for accessibility or requested the presence of a caregiver. In Peru, both in-depth individuals and focus group discussions were conducted. In all settings, the data contained in this paper is part of a larger study exploring the experiences of people with disabilities during the COVID- 19 pandemic (e.g., on livelihoods, access to healthcare). For this paper, we focus on participants’ experiences of preventative measures. Data were collected between 2020–2023 (see Table 1 for details).

**Table 1:** Details of Pandemic Response at Time of Data Collection by Country

Country	Date of data collection	Details of COVID- 19 restrictions in place over the recall period
Bangladesh	April 2021—August 2021	Nationwide periodic lockdowns; mandatory mask-wearing to receive services; border closures; police presence/fines enforce restrictions; closure of schools, other educational institutions, and non-essential businesses
Ghana	May 2021—July 2021	Mandatory mask-wearing (indoor and crowded outdoor spaces); closure of some non-essential businesses (e.g., nightclubs, cinemas); limits on large social gatherings
India	December 2020—March 2021	Movement restrictions; mandatory mask-wearing; school closures; restrictions on social gatherings
Peru	October 2022 – January 2023	Movement restrictions; mandatory mask-wearing; school closures; restrictions on social gatherings during state of emergency In October 2022 the government terminated the state of emergency started in 2020. The lockdowns were discontinued, and the use of masks became optional
Thailand	October 2022 – February 2023	Social distancing; mandatory face mask and hand hygiene; strict travel restrictions and bans on mass gathering; school, and business closures. Replaced by fewer restrictions and a resumption of business and school operation in 2022
Türkiye	May 2021—August 2021	Mandatory mask-wearing in public indoor and outdoor spaces; social distancing; travel restrictions; ban on mass gatherings; closure of some non-essential businesses (e.g., nightclubs, cinemas); school closures; nationwide weekend lockdowns (for periods of 2–3 months)
Viet Nam	December 2021—March 2022	Mandatory mask-wearing in public indoor and outdoor spaces; travel restrictions; closure of some non-essential businesses (e.g., nightclubs, cinemas); restrictions on mass gatherings, school closures
Zimbabwe	May 2021—June 2021	Mandatory mask-wearing (indoor and crowded outdoor spaces); closure of some non-essential businesses (e.g., nightclubs, cinemas); limits on large social gatherings

### Selection and recruitment of participants

Between 17–61 people with disabilities were recruited telephonically and interviewed in each country. Recruitment was also done from the contact lists of previous quantitative surveys and through Organizations of Persons with Disabilities (OPDs) or Non-Governmental Organizations (NGOs). Survey-based recruitment used data from past or ongoing research conducted by in-country research teams, in which they had permission to recontact individuals. Recruitment from OPDs/NGOs involved coordinating with the organisation – whom research teams typically had had past collaborations with – to seek information on people who would be willing to be contacted by either the research partner directly or the OPD/NGO. All lists had basic demographic information (e.g., gender, type of disability, age) and contact details (phone number) of individual. Purposive sampling conducted by in-country research teams was used to promote representation across gender, location (rural/urban), age (children, working age adults, older adults) and disability type (vision, hearing, intellectual/cognitive, speech/communication, physical, and psychosocial/mental health). Potential participants were either approached by the OPD/NGO or the in-country research team.

Wherever possible, adults with disabilities were interviewed directly, and to facilitate this, adaptations were implemented to support their participation. For example, when required, sign language interpretation was available. For individuals who had significant difficulty understanding or communicating even in the presence of available adaptations (e.g., people who are d/Deaf with no knowledge of a formal sign language, or people with severe intellectual/cognitive disabilities), interviews with caregivers or joint interviews with caregivers and the person with a disability, were used. Caregiver interviews were also used for all children aged 10 years and older.

### Data collection

In each country, teams of qualitative interviewers were oriented to the study protocols and received training on qualitative research methods, disability, and research ethics. Interviewers were supervised by an experienced researcher in each country and received in-depth feedback on a sample of 2–5 pilot interviews. Participants were interviewed between 1 December 2020 and 28 February 2023. The interview guides were adapted for each country and piloted prior to use. They contained questions about = various different domains, including experiences of adhering to prevention measures including masking, social distancing, hand hygiene, and accessing vaccines. The depth in which participants discussed prevention measures varied by country due to the time of data collection. Where data collection occurred at the start of the pandemic, more attention was paid to masking, hand hygiene, and social distancing, whereas interviews conducted later (including in 2022), included fewer items on inclusion in prevention.

Most interviews were conducted in the preferred language of participants, although in instances where local dialectal differences made communication on specific topics difficult (notably in Bangladesh), lay interpreters assisted with translation. The interviews lasted approximately 50 min to an hour (the Peru group interviews extended to an hour and three quarters), and were audio and/or video recorded, transcribed, and a portion of the total number of transcripts were translated into English. Due to the restrictions which were in place at the time, interviews were mostly conducted remotely, through Zoom, Teams, Skype, WhatsApp video call or phone call. In Ghana and Zimbabwe, at least some, and in Peru and Thailand almost all the interviews were conducted in-person, because measures at the time allowed for it.

Details of country data collection are shown in Table 1.

### Reflexivity

In each country, a research team was comprised of nationals who had experience in qualitative research and expertise in disability and/or public health. In-country research teams were diverse in terms of characteristics such as gender and in some contexts included persons with disabilities. In-country research teams adapted a semi-structured interview guide for contextual relevance. Data were first analysed at a national level. Cross-country themes were derived through online meetings with all national research teams. A team of male and female researchers, based in the United Kingdom and South Africa, and including persons with disabilities, drafted the initial semi-structured interview guide, coordinated with each in-country team and wrote-up the cross-country themes. These researchers all had experience in qualitative methods and in disability and health research.

### Ethical considerations

Informed consent was obtained from each participant. Consent was written for in-person interviews and given orally and recorded for phone interviews. Caregiver consent was obtained for children below the national age of consent, and for adults with severe intellectual/cognitive disabilities. In these instances, researchers still sought assent from participants if they were able to participate at least somewhat in interviews. Ethics approval for this study was obtained from the London School of Hygiene & Tropical Medicine (22,138 & 28,095) and national review boards in each country: Institutional Review Board, BRAC James P Grant School of Public Health, BRAC University (IRB Reference No. IRB- 22 March’21–008) in Bangladesh; Ghana Health Service Ethics Review Committee (GHS-ERC009/06/20) in Ghana; Institutional Ethics Committee Indian Institute of Public Health Hyderabad (IIPHH/TRCIEC/22/3/2020) in India; Institutional Research Ethics Committee of Universidad Peruana Cayetano Heredia (N° 407–35 - 22) in Peru; Institute for the Development of Human Research Protection Thailand (certificate number IHRP2022085 IHRP No. 065–2565) in Thailand; Koc University Committee on Human Research (2020.306.IRB3.113) in Türkiye; Ethical Review Board For Biomedical Research of Hanoi University of Public Health (No. 427/2021/YTCC-HD3) in Viet Nam; Medical Research Council of Zimbabwe (MRCZ) (No. MRCZ/A/2731) in Zimbabwe.

### Data analysis

Data analysis took place in several stages. In all countries, the local language transcripts for each country were analysed by the country team using deductive thematic analysis. The country team then drafted a report outlining themes and sub-themes. Then, in all countries except Peru and Thailand (where budgetary constraints precluded doing so), a portion of transcripts (10–15 per country) were translated into English and independently coded by four researchers (XH, SH, DG, LMB). This was done to allow for cross-cutting findings to be identified for the dataset as a whole (across countries). For Peru and Thailand, to ensure their inclusion in the cross-country analysis, country teams were invited to purposively translate data which best represented their country dataset. All thematic analysis followed the procedure outlined in Braun and Clarke (i.e. familiarisation with data, developing coding structure and coding data, creating and reviewing themes) [7]. Finally, the findings of the cross-country analysis were written up and compared to the findings from the country-specific analyses. Final themes and sub-themes were discussed by the whole research team through a group Zoom call, country-level meetings, and via comments on the write up of results.

## Results

Details of participants are outlined in Table 2. Overall, 372 people with disabilities and their caregivers were interviewed across the eight countries.^1^

**Table 2:** Participant Details (Persons with Disabilities)^a^

Country	Bangladesh	Ghana	India	Peru	Thailand	Türkiye	Viet Nam	Zimbabwe
Number of participants	60	58	61	69	17	60	23	24
Age of participants								
< 18	33%	35%	26%	50%	–	20%	–	21%
18–64	52%	65%	52%	38%	95%	67%	100%	62%
65 +	15%	–	22%	12%	5%	13%	–	17%
Gender distribution (%)								
Women and girls	50%	59%	33%	50%	58%	45%	47%	54%
Men and boys	50%	41%	67%	48%	43%	55%	53%	46%
Non-binary	–	–	–	2%	–	–	–	–
Disability types (%)								
Cognitive	16.6%	–	29.5%	36%	8%	5%	17%	17%
Communication	11.6%	–	–	–	–	5%	–	–
Hearing	15%	26%	13.11%	16%	23%	17%	7%	21%
Mental health conditions	2.4%	14%	–	9%	15%	8%	17%	–
Physical	16.6%	34%	24.5%	22%	10%	52%	43%	33%
Visual	18.3%	26%	32.8%	17%	10%	22%	17%	21%
Epilepsy	–	–	–	–	–	–	–	17%

For disability type, % can be more than 100% (i.e., people with multiple disabilities were counted in each category, so if a person had a physical and hearing disability, they were in both the physical and hearing categories)

^a^Characteristics of persons with disabilities. For interviews with caregivers, we report on the characteristics of the person with a disability they were reporting about

**Fig. 1:**
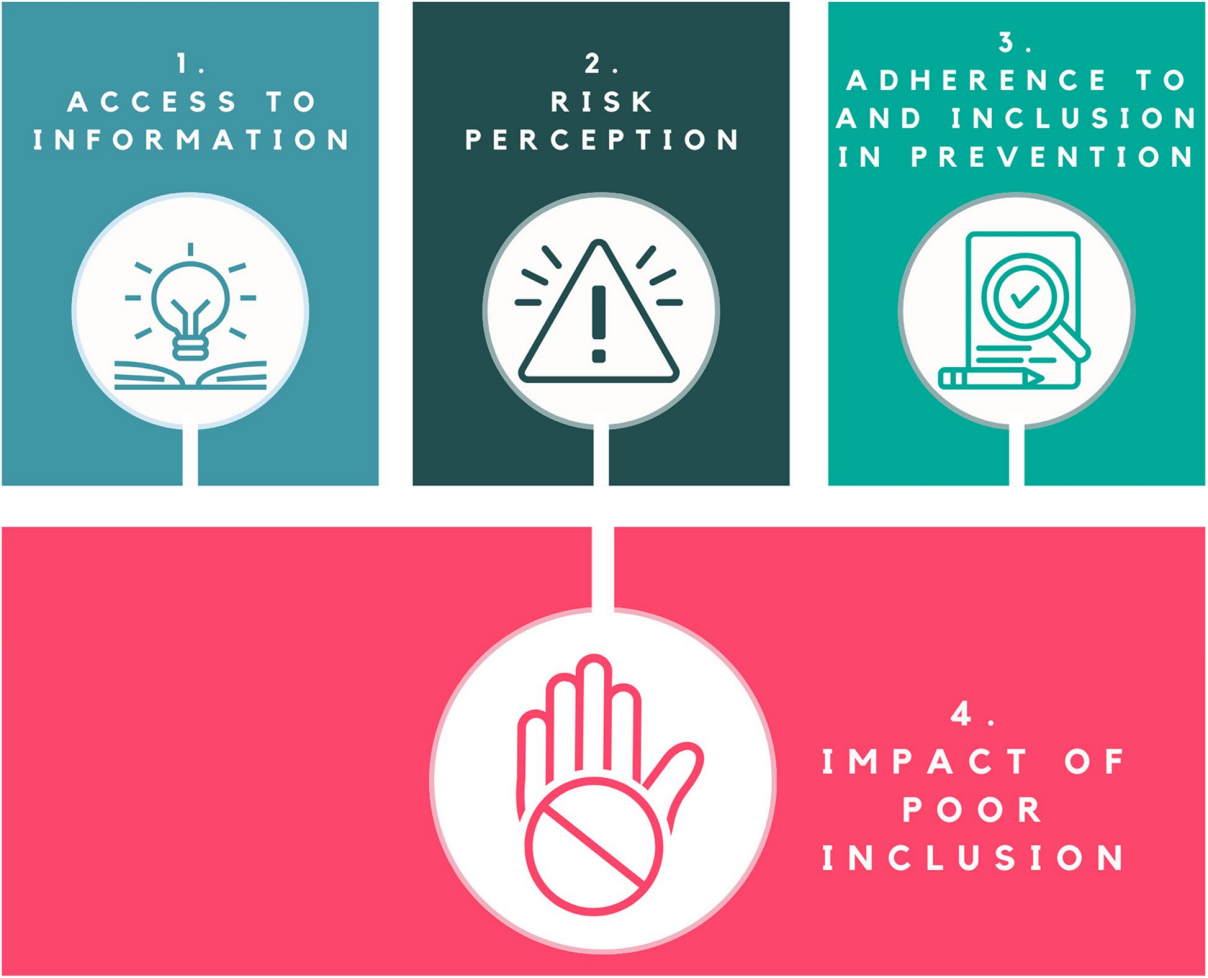
Summary of themes. [Alt text: A figure which shows the four main themes as rectangles, with a round icon representing each theme embedded in the rectangle. The three themes highlighted are: access to information; risk perception; acceptability and inclusion in prevention measures; and impact of poor inclusion]

Themes identified through the thematic analysis are outlined in Fig. 1. They are discussed, along with their subthemes, below.

### Access to information

Almost all participants had at least some basic information about COVID- 19. Children with disabilities, particularly younger children, often were less informed on reasons behind many preventative measures but many at least understood some basics (e.g., need to wear a mask, stay away from others). Participants gained information about COVID- 19 through different sources, including television and print news, radio, OPDs, NGOs, and social media. Many relied heavily on acquiring information through family and friends. OPDs played a key role in this respect. This is reflected in the following from a 15-year-old girl with a hearing disability from Ghana; “I watched a video. Ghana National Association for the Deaf shared video on Covid. [The video taught me] How to wear nose mask, how to wash hands, use hand sanitizers, etc.”

Still, participants reported several challenges in gaining access to needed information about COVID- 19 and related response measures. Access to information was particularly challenging for people with hearing disabilities. In Viet Nam, Bangladesh, Ghana, and Thailand particularly, some participants reported sign language was used in national media and communication materials. For example, the same 15-year-old girl with a hearing disability in Ghana quoted above, described how she’d learnt about COVID- 19 from “schoolteachers and WhatsApp and Facebook”. She also sometimes got information from the news, “[w]hen there is an interpreter on the screen I understand. Ghana TV gives interpreter sometimes for news”, although she acknowledged this interpretation was not available for all broadcasts.

Similarly, in Bangladesh, sign language interpretation was used at the beginning of the pandemic in select national communications but was reported to have gradually decreased in use as the pandemic wore on. Furthermore, many people in the country, people with hearing disability do not use a standardised sign language, and so this format would not have uniformly improved access to information. So, where country-specific sign language variants exist, they still may not have been used by many participants. In Viet Nam, some people with hearing disabilities could not understand the sign language used in communications, either because it was in an unfamiliar version (e.g. Hanoi sign-language was typically used, but other forms of sign language are used in Ho Chi Minh City or Da Nang) or it was difficult to engage with (e.g. interpreter signing too quickly, interpreter too small in embedded frame on the video). In some countries, participants also reported that mask-wearing by sign language interpreters affected understanding:

They [the government] had sign language interpreters in the television wearing masks. You know, d/Deaf people need to see facial expressions to better understand sign language. We couldn’t properly understand sign while interpreters were masked. That’s why d/Deaf people stop watching TV.

[40-year-old man with a hearing disability from Thailand].

Further, people with low literacy faced difficulties reading print materials. Many people with onset of disability during school going years were excluded from education, and so often did not have the level of literacy required to engage with written information leaflets concerning COVID- 19. Much of the COVID- 19 information, education and communication documents and news used technical language, such as “symptoms of the respiratory system” or “antiviral drugs”, that were difficult to understand for people with intellectual disabilities and those with limited schooling.

I went to school but learnt nothing, just was present in the class…I did not know much about the lessons…So, now I just know some words, but some I don’t. I can read some news about COVID, but some I can’t. I can read very short pieces of news with very short sentences only. Long pieces of news or long sentences are out of reach. I can’t understand long pieces of news or long sentences.

[24-year-old man with a physical disability from Viet Nam].

People, especially children, with intellectual disabilities sometimes faced difficulties understanding information about COVID- 19. As one caregiver from Ghana noted about her daughter, a 14-year-old with an intellectual disability: ‘She is observing the protocol, but she doesn’t really understand the reason why…. she wants to be removing [her face mask] within a short time when she wears it.’ Some caregivers were able to communicate information about mask-wearing and other protocols through demonstration and frequent reminders. Caregivers also reported resorting to scare tactics to convince children and adults with intellectual disabilities to adhere to regulations in the absence of understanding. For instance, a caregiver of a 34-year-old man with an intellectual disability in Türkiye explained “Because he for 2 months he got blood poisoning, so I always tell him if you don’t wear the mask, I’ll take you back to the hospital, so he gets scared and put the mask, else he doesn’t know”.

Finally, misinformation was a challenge, particularly in the context of vaccine hesitancy. Most participants reported that they either had taken the vaccine or planned to take it. However, some participants, particularly in Ghana and Viet Nam, reported being nervous of side effects or shared misinformation about vaccines during the interviews. These beliefs resulted in reluctance to get vaccinated or take subsequent doses of the vaccine if they had experienced side effects. Scepticism about the effectiveness of the vaccines for people with disabilities were also reported. For example, several d/Deaf participants in Ghana reported seeing videos about people dying or experiencing negative side effects of vaccination. These videos were available in accessible formats (i.e., with sign language) and so were very accessible to people with disabilities:

[The vaccine] is not good.…Already we are disabled and if the vaccine has something bad it will kill us faster than the abled ones… Yes, and I saw a video that deaf people in Africa must not take vaccine. They sign in the video like “deaf in Africa no vaccine”. I don’t know why…. Honestly, I am afraid. Because I hear some people get sick and I saw a video—some lady died when she received the vaccine.

[38-year-old man with a hearing disability from Ghana]

### People with disabilities’ perception of their risk of COVID- 19

Perceptions of individual risk to COVID- 19 infection varied amongst participants, but many felt they were at a heightened risk of becoming infected or having worse health outcomes (e.g., severe symptoms, hospitalization, death) compared to people without disabilities. Most of these perceptions of increased risk were attributed to either their disability or underlying health condition, their daily life activities, or challenges in following preventative measures (see Sect. 1.2 below). For example, people with vision disabilities and mobility limitations noted difficulties maintaining social distancing, and people who were reliant on personal carers found it difficult to implement social distancing measures, which placed them at risk of infection. As one 50-year-old man with physical disability from Thailand explained, “People with severe physical disabilities can't be parted from their personal assistant. Some got infected from their caregivers. Caregiver needs to go outside to the market and come back home to bathe the person with a disability.”

Some participants also worried that they would suffer worse health outcomes if they did become infected with COVID- 19. In particular, older adults and people with certain underlying health conditions (e.g., diabetes, hypertension) felt they were more at risk, given the well-publicised links between age, these health conditions, and COVID- 19 mortality. For example, a 58-year-old man with a physical disability in Türkiye shared that he was “afraid of infection because of [his] age, [he would] not be able to resist the disease”. A 67-year-old man with a physical disability from Zimbabwe similarly explained:

I am at risk because I no longer walk on my own, hence I cannot go in gatherings. I always ask people when they come back from such places to tell me what has been said, because l am scared of going to crowded areas as I am weak. Those people might bring the infection back to me.

Other people felt that having a disability in and of itself led to a higher risk of poor health outcomes from COVID- 19, even if there were no known links with their disability and COVID- 19 morbidity and mortality. For example, a caregiver of a 6-year-old child with autism in India described how her son was more at risk of COVID- 19 than others because “all the autistic kids don’t have immunity… they are already weak”. Similarly, a young man from Viet Nam with hearing and speech disabilities explained how people with disabilities like his “may look healthy like me, but their bodies do not work functionally from inside so that they cannot speak and listen.”

Others felt that their risk was no different compared to people without disabilities. As a 45-year-old man with a hearing disability from Türkiye explained, “Every disease is in the hand of God. Even if I get infected, I don’t think it would have that big of impact on my health or my family’s health.” A few participants indicated scepticism that COVID- 19 was even present in their community or felt that warnings about the disease were exaggerated.

Overall, there were many instances in which people with disabilities’ and/or their caregivers’ perception of their increased risk resulted in their being kept indoors and isolated, for fear of infection. As one caregiver of an 84-year-old man with a cognitive disability from Peru noted, this social isolation was often borne out of fear: “During the pandemic, because of the confinement… he could not go out because we were afraid… that he would catch the disease.”

### Adherence to and inclusion in preventive measures

Almost all adults with disabilities included in this study were at least somewhat aware of the main preventative measures, including hand washing, mask-wearing, vaccination and requirements to follow social distancing and lockdown mandates. Many children were also aware of these measures, but their caregivers were influential in determining adherence, more so, perhaps, than individual levels of knowledge. In many countries, participants were willing to adhere to most of the prevention measures that they were aware of. Many saw prevention measures as a set of rules laid out by government for the public good and as a way of reducing their own risk of infection. A woman in Türkiye expressed these sentiments in relation to mask-wearing:

Generally, the mask is, that’s it. We accepted the matter of fact. Like this what it is, this is what happens, that’s it. This is something for our benefit, it’s not something that’s against us. We followed it and were convinced by it. If you accept it or not, this is something that has to happen. We are obligated to follow it.

[52-year-old woman with a hearing disability in Türkiye].

However, in other countries, motivation for adherence were different: several participants in Ghana shared that fear of being penalised by the state for not following rules had played a role in shaping adherence to prevention measures. In Viet Nam, one participant indicated he was ‘made’ or compelled to get vaccinated. She explained:

I did [have concerns about the COVID- 19 vaccine]. I wondered about the vaccine’s effectiveness and side effects among people with hearing and speaking disabilities. Our bodies are incomplete and cannot function normally as people without hearing and speaking disabilities.

[30-year-old woman with hearing and communication disabilities from Viet Nam].

Still, acceptability differed across recommended preventative measures. In some cases, this was because prevention measures interacted with people’s disabilities to cause pain and discomfort. One 25-year-old woman with albinism from Zimbabwe described how"the sanitizers that I have used have burnt my skin”. However, in most instances, engagement in prevention measures was shaped by the fact that people with disabilities faced additional barriers to following them, including lack of accessible information and adaptations to standard measures. Factors affecting acceptability and inclusion in preventative measures are described below.

### Accessibility of services

Physical accessibility of health facilities was a reported challenge for many participants across countries, including when people went for COVID- 19 vaccination or testing and treatment. In Thailand, one 50-year old man with a physical disability explained that they *“Community isolation [facilities] were not designed for us. Wheelchair users can’t use the toilet.”* Long wait times and needing to stand in crowded lines when going for vaccination or testing were reported in several countries, including in Bangladesh and Ghana.

Also, I will have to wait in a queue if I go to get vaccinated or do a Corona test…The Government should have prioritised disabled persons so that they didn’t have to stand in a queue and that they would be provided with support immediately. It’s not like it would have cost extra money.

[32-year-old man with a vision disability from Bangladesh].

Further, inaccessibility of communication in health facilities was a challenge for some participants, particularly people with hearing disabilities.

[T]wo months ago, I got COVID. The hospital separated the COVID screening zone outside the building [to prevent spread of infection]. There was nothing on-screen for queue numbers [numbers in the queue are called out]. I just sit there and wait endlessly. I don’t know whether I had missed my cue or not.

[50-year-old man with a hearing disability from Thailand].

Finally, there were a few reports of a lack of support from health care workers which appeared to stem from inadequate training on disability inclusion, and possibly inadequate training in respect of the pandemic response in general:

I asked the health professionals at the vaccination spots one question only: ‘What is the vaccine I will get?’. They said “the Chinese vaccine” for the first and second doses, and “Astra”, ah no no, “Pfizer” for the third dose. They also told me to read more in the vaccination certificate and contact them for more information if needed, but I can’t see and can’t read obviously.

[72-year-old woman with a visual disability from Viet Nam]

In Peru, one participant explained how restrictions on accompaniment to health facilities and in procedures negatively impacted them, as they usually relied on a personal assistant:

I need a lot of support for going out, I don't usually go out alone, because I don't know where I am, it is hard for me to find my way in the streets and besides, my anxiety is too great to be able to be alone in the street… the most complex was that [in the health centre] they only let one person in and... well, they wouldn't let me in with any companion and for me that was very, very complex actually, because it makes me very anxious to be alone in places I don't know.

[32-year-old non-binary person with autism from Peru].

Still, it is important to highlight that in some countries adaptations were put in place to improve inclusion for people with disabilities. This seemed to be particularly the case with vaccination. In Viet Nam, home-based vaccination was offered for people with mobility limitations, although this adaptation was put in place only once vaccine roll-out was well under way and most people had already received the recommended course. In Peru and Ghana, home-based vaccination was also rolled out in some areas. As a 30-year-old man with intellectual disability from Ghana explained, an outreach nurse had come to his house to administer the vaccine, “so it was very easy [to get vaccinated].”

When vaccination first started in Türkiye, there were a limited number of vaccines, and so the government started the vaccination process for elderly people and people with disabilities first, and both of these groups were targeted with calls and outreach. As one participant explained:

I didn’t know anything about the vaccines, but they called me a couple of weeks ago and told me that my turn in the vaccines has come and I took it then they called after some weeks and told me to go again and I took another one then I asked if they need anything else from me and they told me no we are done. I was the first one to get vaccine from my family and I did not get any side effects or feel anything.

[62-year-old man with a physical disability in Türkiye]

Similar prioritisation of the elderly and people with certain types of disabilities was reported in Peru.

#### Lack of adapted guidelines

Much of the information about how to prevent COVID- 19 that was received by participants did not offer adaptations reflecting the needs and concerns of people with disabilities. As such, participants often faced difficulties in following guidelines.

Importantly, many participants required assistance from family, informal caregivers, and other community members for daily life activities. People with mobility and visual limitations in particular often relied on physical touch of surfaces or assistance from others to move around. This required way of operating was often not in line with recommended social distancing and hygiene practices. As one participant noted:

Suppose I am at a market now. And I am going through the market for some reason. Okay? Other people can go past others without bumping into them, but in my case, I get bumped into others. Suppose someone is coughing or sneezing. If there are other people around him [someone without a vision disability], they may move away immediately, but I can’t. As it happens, sometimes, people cough on me.

[32-year-old man with vision disability from Bangladesh]

Some participants also noted difficulties using PPE as recommended. For instance, people with upper body limitations and people with vision disabilities noted that masks were difficult to manipulate. Further, d/Deaf people noted that masks interfered with communication, particularly for people using lip reading.

Additionally, caregivers of children and adults with intellectual disabilities faced difficulties explaining protocols and ensuring compliance. For example, the mother of a 26-year-old woman with intellectual disability in Bangladesh explained how she had explained repeatedly protocols to her daughter “but she can’t remember it. Whenever she meets her friends, she hugs them.” They also worried about how they would know if their child or other family member with a disability had COVID- 19 due to difficulties describing their symptoms. For example, the caregiver of a young woman with hearing and speech disabilities in India described:

I have to be very careful about her [daughter with disability]. I don’t allow her to go outside. She can do whatever she wants to do inside the house… if she has suffered from the disease, she would not be able to tell us easily.

[Caregiver of a 23-year-old woman with hearing and communication disabilities from India]

#### Financial barriers

Many participants noted that buying PPE such as masks, hand sanitiser, and soap created a substantial cost burden which they struggled to bear. This was particularly the case for people living in poverty.

Eiii! [Buying a mask] really brought financial issues. It is now that the price has reduced a bit but at first it was very expensive. The price for single one was GHS 1.00, so even if you are three people in a household, you need to spend GHS 3.00 at a time [US$0.30].

[39-year-old woman with a physical disability from Ghana].

For some participants, this additional cost was amplified because of disability-related extra costs and having multiple competing demands on very limited resources. As one man explained:

There is no money, my medication for my eyes needs US$3 at the pharmacy, there is no way I will ask for a sanitiser whilst I have problems with my eyes. I would rather be fighting for my eyesight so that I will not be blind forever. I was advised not to default on my medication, so I’m not supposed to miss the medication.

[43-year-old man with visual disability from Zimbabwe].

Some participants reported receiving food or other essentials from OPDs, NGOs, and other charitable support structures (including through community kitchens or schools). Further, some participants received top-ups to existing or new social protection entitlements. In Peru, the government delivered unconditional emergency cash transfers to poor and vulnerable households at various stages during the COVID- 19 pandemic.

In Peru, several participants spoke about the value of COVID- 19 cash transfers in alleviating some of the financial stress associated with the pandemic and associated costs. As one caregiver of an 84-year-old man with cognitive disability from Peru noted,"…the ‘bonos’ [cash transfers] were for my dad's expenses, to buy his medicines, his little things, his vitamins… it has been a help… we really needed, because we were not working".

In Türkiye, most participants were previously receiving a regular cash transfer from the Red Crescent due to their refugee status. However, the value of the transfers was often seen to be too low. One 58-year-old man with a physical disability explained that even though his Red Crescent transfer increased from 150 lira to 250 (US$6 to US$9) during COVID- 19, it was still “not enough for anything”. Overall and across countries the coverage of these entitlements was low and insufficient in the context of rising living costs and reduced earnings due to the pandemic.

### Impact of poor inclusion

The lack of inclusion in preventative measures left people with disabilities in a difficult position of deciding how to best protect their health and the health of others. As a result, several participants implemented additional measures to protect their health, particularly if they felt their risk of exposure or negative consequences of infection were particularly high. These measures often went beyond the recommended guidance (e.g., double/triple masking, sanitizing face masks, additional hygiene practices):

Whenever back at my home, I’ll disinfect or wash my hands by [a local sanitizer brand]. Even at home, I’ll wash my hands if I feel dirty. Wash, wash, wash [...]. My health is not as good as [people without disabilities], so I have to wash my hands more carefully to eliminate any infectious risks. My mother-in-law has told me that I will make my hands’ skin fragile with that washing style, but I feel insecure if not doing so.

[35-year-old woman with a physical disability from Viet Nam]

Several participants, particularly caregivers of children with disabilities, reported that they had severely reduced social contacts to prevent exposure. For example, the caregiver of a 6-year-old boy with cerebral palsy from India explained how her son had not left the house for over half a year due to her fears of his vulnerability to COVID- 19:

I am not taking him out of the house for anything; he is in the house, so this [COVID- 19 pandemic] has affected us and him so badly. He loves going out, he loves meeting people especially people who he knows very much, I mean all the near and dear ones… still he was not being taken out for like 6–7 months or so.

While these measures may have been effective at preventing infection, they had serious negative impacts on people’s mental health due to the prolonged isolation, lack of stimulation and fear.

## Discussion

Overall, almost all participants across the eight countries were aware of COVID- 19 and at least some measures to prevent infection. Still, there were gaps in comprehensive knowledge, due primarily to a lack of accessible information. Further, despite being aware of preventative measures and willing to adhere, many found it difficult to comply without adaptations or support. As a result, people with disabilities either were unable to follow adequate prevention measures or took additional steps to protect their health, including long-term isolation. The impacts of these exclusions were compounded by additional challenges faced by people with disabilities during the pandemic in respect of access to healthcare [22], and education [23].

The present study highlights several factors that affected the ability to follow preventative measures. First, accessible information was critical but often lacking, as has been highlighted in other studies [9, 56]. For example, access to information for people with certain types of disability, particularly hearing and vision disabilities and intellectual disabilities, was restricted owing to limited availability of sign language interpretation and communication in easy-to-read or verbal formats. There were several attempts to produce information in accessible formats, but these were often limited in reach, offered inconsistently, and still posed obstacles to transmitting or receiving information (e.g., differences in regional sign languages, interpreters wearing masks or too small to see on screen). Other communication barriers included low literacy amongst participants, a factor that may be more pronounced amongst people with disabilities given their exclusion from education [38]. Further, age-appropriate and accessible information is critical for children with disabilities – in their absence children with disabilities were less able to protect themselves and faced increased fear, confusion and isolation. Caregivers, healthcare workers, school staff and other key workers also require information that is relevant for providing safe care and support to children and adults with disabilities in the context of COVID- 19.

This research also revealed that social distancing and other mandates were difficult to adhere to for some people with disabilities – particularly those requiring care and support. This finding emphasises the need for adapted COVID- 19 guidelines that account for specific needs and concerns of children and adults with disabilities. Accessibility of health facilities was also a major barrier, particularly for vaccination and testing. Issues of health service accessibility have pre-dated the pandemic, and were then exacerbated as services became over-strained [18]. Nonetheless, there were calls for guidance tailored to people with disabilities from the start of the pandemic. These calls focused on key issues such as training of healthcare workers on disability; priority COVID- 19 or home-based vaccination for persons with disabilities; tailored advice to caregivers and other key personnel to promote the safety of people with disabilities; and disability disaggregated COVID- 19 data [2, 31, 44, 48]. Amongst countries belonging to the United Nations, 50% published recommendations specifically for the prevention of COVID- 19 amongst people with disabilities [36]. However, often the onus of protection was put on individuals, without providing support to make these measures feasible nor recognise the diversity amongst people with disabilities when providing adaptations [12, 36, 45, 55].

Finally, the cost of purchasing hygiene products (e.g., masks, soaps, sanitisers, PPE) and complying with lockdowns was prohibitive for many people with disabilities and their families. Even before the pandemic, people with disabilities experienced disproportionately higher levels of poverty, and had to contend with disability-related extra costs [4, 42]. Similarly, before and during the COVID- 19 pandemic, people with disabilities generally have lower access to adequate water, sanitation, and hygiene (WASH) within their households and face greater barriers to independently using them [5, 37]. The pandemic and consequent lockdowns further constrained the economic situation of people with disabilities [17, 20, 46]. These findings imply that in addition to public health awareness, pandemic prevention policies need to account for multidimensional poverty experienced by people with disabilities and their households.

From the study countries, there were some examples of good practice identified in respect of inclusion of persons with disabilities. For example, some countries implemented measures such as home visiting for vaccination in Ghana and top-ups to cash transfers in Türkiye. In other countries, such as Colombia, Mexico, and Argentina, for instance, national interventions were rolled out to support inclusion, many with a focus on access to information. In Colombia, the government made Job Access With Speech (JAWS) screen-reading software and ZoomText text magnifying software free to download and widely available for people with vision disabilities [53]. In Mexico, the government developed specific accessible communication guides for people with physical, hearing, intellectual, psychosocial, and visual disabilities [53]. Finally, in Argentina, a new WhatsApp service was created to answer questions and attend to emergencies, and included a free sign language telephone number to answer questions about COVID- 19 [53]. Similarly, in Peru all COVID- 19 information had to be provided in accessible formats, and community networks were established to identify and support people with severe disabilities [45]. Further research may be needed to understand the reach of these other measures, and how effective they were in practice for people with disabilities in these countries.

In all study countries, civil society organisations—comprising of NGOs and OPDs —stepped in to fill the void left by tenuous state responses (a finding which is echoed in other countries [21, 35, 40]). These civil society organisations were instrumental in translating COVID- 19 information into accessible formats, distributing hygiene kits, food packages, and in some cases cash support, and advocating for greater consideration of people with disabilities in prevention and response measures [11]. The coverage of OPDs, however, was mostly restricted to their existing members and many were constrained due to severe shortages of resources. A recent study based on survey data from nine countries also found that generally less than 10% of people with disabilities are part of OPDs; women, older people, people with intellectual and developmental disabilities and people living in poverty and in rural areas are much less likely to be aware of or be affiliated to an OPD [3]. Responses to future pandemics should, therefore, focus on partnering with and extending the capacity of OPDs, and formulate alternative ways of including people with disabilities, who experience multiple layers of marginalisation due to their intersecting identities. This approach has proven successful in other emergencies, including humanitarian crises, natural disasters and in response to other disease outbreaks [24, 28, 29].

### Strengths and limitations

Similar methodologies were employed across eight different countries, with an unusually large sample and geographic range for a qualitative study. Furthermore, coding of transcripts in local languages, and double coding of some in English, contributes to the reliability of the findings. Finally, the inclusion of diverse voices of people with disabilities (e.g., different types of disabilities, gender, location) increases the applicability of the findings.

However, some limitations must also be noted. For instance, interviews were mostly conducted remotely, through Zoom, Teams, Skype, WhatsApp video call or phone call because of COVID- 19 restrictions in place during data collection. While necessary to protect participants’ and research team members’ health, this had consequences. For example, people with hearing disabilities faced some barriers in participating – sign language was available, but people needed smart phones to engage in a video interview, which many did not have. Additionally, there are well-documented differences between digital and in-person data collection of qualitative data [51]. Finally, digital access is considered a social determinant of health, and by collecting data using digital means, there is a risk of compounding disparities in participation in research by excluding marginalised groups who do not have access to phones or computers [13]. Further, some countries recruited through OPDs. This means that the samples are not as representative as they might have been, because there are documented issues with the representativeness of these organizations [3]. Finally, it is possible that in countries where interviews occurred later in the course of the pandemic, particularly Thailand and Peru, that recall bias may have influenced findings, and this affected the comparability of the findings from these countries with those stemming from the others.

## Conclusion

Globally, people with disabilities were excluded or inadequately included in COVID- 19 pandemic response measures [43]. This exclusion compounded barriers and exclusions experienced by people with disabilities prior to the start of the pandemic. Our work echoes previous findings on disability inclusion in the 2015 Ebola crisis response, which also found that pre-existing inequalities were compounded during the outbreak [28, 29]. It is notable that despite diverse contexts in the study countries, people with disabilities, to a large extent, had similar experiences with prevention and response measures, pointing to the role of wider structural processes in hindering people with disabilities’ equal participation in society.

Going forward there is a need for global consensus-building to develop recommendations for disability-inclusive emergency preparedness and response systems. Moreover, this research raises important questions about the lives of people with disabilities in the ‘post-pandemic’ era: if people with disabilities were markedly excluded from pandemic responses, are they being similarly excluded from recovery efforts?

Overall, this paper highlights the need not only to look back and learn from the COVID- 19 pandemic as one example of an emergency response marked by poor disability inclusion, but also to look to the future and put in place strategies for planning and preparedness to be inclusive. Furthermore, recovery measures must be examined and evaluated through the lens of disability inequalities.

## Data Availability

The datasets generated and/or analysed during the current study are not publicly available due to the difficulty of anonymising qualitative interview transcripts and the risks which archiving pose to study participants but are available from the corresponding author on reasonable request.

## Footnotes

1 The vast majority of participants were people with disabilities themselves. However, in some instances (discussed above), a caregiver completed the interview on behalf of a person with a disability, or together with them. In these cases, the demographic information of the person with a disability about whom they were responding, is included in the table below.
